# Attachment anxiety predicts depression and anxiety symptoms following coronary artery bypass graft surgery

**DOI:** 10.1111/bjhp.12191

**Published:** 2016-10-06

**Authors:** Tara Kidd, Lydia Poole, Amy Ronaldson, Elizabeth Leigh, Marjan Jahangiri, Andrew Steptoe

**Affiliations:** ^1^ Department of Epidemiology and Public HealthUniversity College LondonUK; ^2^ Department of Cardiac SurgerySt. George's HospitalUniversity of LondonUK

**Keywords:** depression, anxiety, coronary artery bypass graft, attachment

## Abstract

**Objective:**

Depression and anxiety are associated with poor recovery in coronary artery bypass graft (CABG) patients, but little is known about predictors of depression and anxiety symptoms.

**Design:**

We tested the prospective association between attachment orientation, and symptoms of depression and anxiety in CABG patients, 6–8 weeks, and 12 months following surgery.

**Method:**

One hundred and fifty‐five patients who were undergoing planned CABG surgery were recruited. Patients completed questionnaires measuring attachment, depression, and anxiety prior to surgery, then 6–8 weeks, and 12 months after surgery.

**Results:**

Attachment anxiety predicted symptoms of depression and anxiety at both follow‐up time points, whereas attachment avoidance was not associated with depression or anxiety symptoms. The findings remained significant when controlling for baseline mood scores, social support, demographic, and clinical risk factors.

**Conclusion:**

These results suggest that attachment anxiety is associated with short‐term and long‐term depression and anxiety symptoms following CABG surgery. These results may offer important insight into understanding the recovery process in CABG surgery.

**Statement of contribution**

***What is already known on this subject?***

- Depression and anxiety symptoms are twice more likely to occur in coronary artery bypass graft (CABG) populations than in any other medical group.
- Depression and anxiety are associated with poor recovery following cardiac surgery.
- Predictors of depression and anxiety in CABG patients have been underexplored.

***What does this study add?***

- This study highlights the importance of close interpersonal relationships on health.
- Attachment anxiety was prospectively associated with higher levels of depression and anxiety.
- These results add to understanding mechanisms linked to recovery following CABG.

## Background

Many studies have shown a strong relationship between depression and the development and progression of coronary artery disease (CAD), independently of established risk factors (Blumenthal *et al*., 2003; Pignay‐Demaria, Lespérance, Demaria, Frasure‐Smith, & Perrault, 2003). It has been estimated that a depressed person is 1.5 times more likely to develop CAD than that of an otherwise healthy person (Bradley & Rumsfeld, 2015). Depression is also likely to occur twice as often in cardiac populations than any other medical group (Whooley, 2006), with highest rates reported in patients with unstable angina or those awaiting coronary artery bypass surgery (Blumenthal *et al*., 2003). Coronary artery bypass graft (CABG) surgery is now one of the most common cardiac procedures in the United Kingdom and United States with over 500,000 procedures carried out per annum (Townsend *et al*., 2012). The benefits of CABG on physical health are well documented; however, there remains significant variation in recovery which has been partly attributed to depression (Burg, Benedetto, & Soufer, 2003; Connerney, Shapiro, McLaughlin, Bagiella, & Sloan, 2001). Therefore, identifying those who are susceptible to depression has never been more important, with prevalence rate estimates ranging from 27% to 61% (Pignay‐Demaria *et al*., 2003; Tully *et al*., 2011).

Considerable evidence details the association between depression and poor recovery both in the immediate period following CABG surgery and in the longer term (Burg *et al*., 2003; Saur *et al*., 2001). Markers of short‐term recovery, including longer in‐hospital stay following surgery, pain, graft site and wound infection, have been linked with depression (Doering, Moser, Lemankiewicz, Luper, & Khan, 2005; Poole, Kidd *et al*., 2014; Poole, Leigh *et al*., 2014). Similarly, indices of long‐term recovery such as re‐hospitalization rates, future cardiac events, reductions in quality of life (QoL), and increased mortality have also been associated with depression (Blumenthal *et al*., 2003; Connerney *et al*., 2001). The proposed mechanisms underlying the association between depression and CABG outcomes are complex, comprising biological, behavioural, and social pathways (Joynt, Whellan, & O'Connor, 2003; Lett *et al*., 2004).

Anxiety has also been tentatively linked to the development and prognosis in patients with CAD, both independently and in conjunction with depression (Oxlad, Stubberfield, Stuklis, Edwards, & Wade, 2006; Pignay‐Demaria *et al*., 2003; Roest, Martens, de Jonge, & Denollet, 2010). The interpretation of findings is complicated by variations in the type of anxiety evaluated, which can include state/trait anxiety, generalized anxiety disorder (GAD), and social anxiety (Tully, Baker, & Knight, 2008). Nonetheless, it has been reported that acute pre‐operative levels of anxiety predict greater adverse outcomes following surgery, including a doubled risk of re‐admission following surgery (Connerney *et al*., 2001). Furthermore, pre‐operative anxiety in CABG patients has been significantly associated with increased post‐operative complications and higher incidents of further cardiac events (Oxlad *et al*., 2006; Pintor *et al*., 1992).

Surprisingly, relatively little is known about the predictors of depression and anxiety in CABG patient populations. High prevalence of mood disorders in cardiac patients is not explained by disease severity or cardiac‐related impairments alone (Frasure‐Smith, Lespérance, & Talajic, 1993; Gonzalez *et al*., 1996). There is evidence that psychosocial factors such as social support and socio‐economic status can predict post‐operative mood (Barth, Schneider, & von Känel, 2010; Poole, Leigh *et al*., 2014); however, currently, pre‐operative depression and anxiety remain the strongest and most consistent predictor of post‐operative levels (McKenzie, Simpson, & Stewart, 2010; Pirraglia, Peterson, Williams‐Russo, Gorkin, & Charlson, 1999). Moreover, pre‐operative depression has been found to predict post‐operative depression in CABG patients up to 5 years following surgery, with reports of upwards of 20% of patients still experiencing depressive symptoms (Koivula, Halme, & Åstedt‐Kurki, 2010). As depression and anxiety are often considered disorders that arise from dysfunctional emotional or affect regulation (Campbell‐Sills & Barlow, 2007; Nolen‐Hoeksema, Wisco, & Lyubomirsky, 2008), perhaps examining those factors associated with affect regulation may be providential. Clearly, identifying factors that predict depression and anxiety symptoms over and above pre‐existing mood is critical to understanding patterns and variation in symptom reporting.

Attachment theory is a major theoretical framework used in understanding individual differences in affect regulation (Mikulincer & Shaver, 2012). Briefly, attachment is part of an evolutionary system designed to promote the survival of an infant, by reinforcing basic capacities to respond to a potentially threatening situation. In infants, both emotional arousal and physiological arousal are initially regulated by the response of the caregiver during a threat. Repeated interactions over time result in the infant learning to regulate themselves independently of the caregiver. Schemas are formalized consisting of expectations related to threat, strategies used to express or inhibit emotions, as well as initiating a physiological response to a perceived threat (Hazan & Shaver, 1987).

Attachment can be described in terms of two independent dimensions, namely attachment anxiety and avoidance (Brennan, Clark, & Shaver, 1998). Individuals who are high in attachment anxiety have a negative view of self, and fear abandonment due to receiving inconsistent care in their formative years. They utilize hypervigilant strategies aimed at maximizing proximity to their attachment figure, typically a romantic partner in adults, to manage distress; however, this means that emotions tend to be augmented, and fears that care will not be received increase the overall experience of negative affect. Individuals high in attachment avoidance have a positive view of self, and tend to minimize feelings of distress and direct attention away from potential threat by maximizing autonomous behaviour strategies. This behavioural pattern is in response to receiving non‐responsive care in infancy, where independence was reinforced. Depending where an individual is located on these scales will determine the propensity to see a threat (affect reactivity) and the strategies used to regulate distress (affect regulation), which are considered the core function underlying adult attachment (Bowlby, 1969; Feeney, 2000; Mikulincer, Shaver, & Pereg, 2003; Pietromonaco, Barrett, & Powers, 2006).

Studies have shown that the attachment system is activated during perceived or actual threat to the self which can fall into three categories: personal threat (hunger, pain, death), environmental threat (challenging situation), and relational threat (separation from partner; Bowlby, 1969; Diamond, Hicks, & Otter‐Henderson, 2008; Kidd, Hamer, & Steptoe, 2011 **)**. The idea of studying attachment in relation to health, a personal threat, is a relatively recent phenomenon (Maunder & Hunter, 2001). While the focus has been on chronic health outcomes, there is some evidence that surgical procedures are also likely to initiate the attachment system (Szymczak, 2004). Although, to our knowledge, this has yet to be tested in a cardiac population, it is plausible that cardiac surgery may constitute one or all types of threat activating the attachment system; if that is the case, then it may allow some insight into individual variation in depression and anxiety symptoms in CABG patients.

Extensive empirical research has demonstrated strong support for the role of attachment in affect reactivity and regulation. As one would expect, those who have insecure attachments, especially those who are high in attachment anxiety report more intense emotions (Collins & Read, 1990), greater fluctuating emotions (Hazan & Shaver, 1987), higher levels of anxiety (Hankin, Kassel, & Abela, 2005), and depression symptoms (Shaver, Schachner, & Mikulincer, 2005) than avoidant individuals. However, studies have largely examined healthy adults in laboratory situations and little is known about the role of attachment in symptom reporting of depression and anxiety in different patient populations (Ciechanowski, Sullivan, Jensen, Romano, & Summers, 2003; Maunder, Lancee, Hunter, Greenberg, & Steinhart, 2005).

The aim of this study was to examine whether attachment may predict depression and anxiety symptoms at short‐term follow‐up (6–8 weeks) and in the longer term (12 months) following CABG surgery over and above established risk factors, including baseline values, SES, and social support.

## Method

### Participants

The analyses were carried out using data from the Adjustment and Recovery after Cardiac Surgery (ARCS) Study, a longitudinal study of socio‐economic, psychosocial, and biological risk factors for recovery after CABG surgery. Two hundred and sixty‐five participants were recruited at a pre‐surgery assessment clinic from a UK hospital, which was on average 29 days prior to surgery. Baseline questionnaires were administered, and cognitive function was assessed during this clinic visit. Follow‐up data were collected 6–8 weeks following surgery and at 12 months using postal questionnaires. For this study, we report only the depression and anxiety outcomes at these time points. Only patients who had fully completed all the relevant baseline questionnaires and depression and anxiety questionnaires at 6–8 weeks and 12 months, who scored 19 or above on the Montreal Cognitive Assessment (MoCA; Nasreddine *et al*., 2005), had complete covariate data, and were undergoing elective CABG surgery (±valve replacement) were included in the analysis. One hundred and fifty‐five patients were eligible to take part in this study (135 males, 20 females, age range 44–90 years). Further detail on recruitment and retention can be found in Poole, Kidd *et al*. (2014) and Poole, Leigh *et al*. (2014). All procedures were carried out with the written consent of the participants. Ethical approval was obtained from the National Research Ethics Service.

### Measures

#### Clinical and sociodemographic measures

Cardiovascular history and clinical factors were obtained from clinical notes. Clinical risk was assessed using the European System for Cardiac Operative Risk Evaluation (EuroSCORE; Nashef *et al*., 1999). EuroSCORE is a composite measure of procedural mortality risk based on 17 factors comprising patient‐related factors (e.g., age and sex), cardiac‐related factors (e.g., unstable angina, recent MI), and surgery‐related factors (e.g., surgery on thoracic aorta). Items were scored in accordance with the ‘logistic EuroSCORE’ method to generate a percentage mortality risk estimate. This uses a formula which incorporates a weighted score for each component, computed to maximize accuracy of multivariate prediction; further details of the scoring method can be found of the EuroSCORE website (www.euroscore.org/logisticEuroSCORE.htm). Participants detailed their medications prior to surgery and total number of long‐standing illnesses (chronic disease burden). Socio‐economic status was assessed using yearly household income in five categories ranging from <£10,000 per year to >£40,000 per year. Body mass index (BMI) was assessed at the pre‐operative clinic appointment and calculated using the standard formula (kg/m^2^).

#### Depression

Symptoms of depression were measured using the Beck Depression Inventory (Beck & Steer, 1987) at baseline and follow‐up. The BDI is a 21‐item multiple response measure and examines somatic and non‐somatic symptoms of depression. The scores vary from 0 to 63 points, and patients with scores lower than 10 can be regarded as asymptomatic in the general population. Higher scores indicate higher distress. Cronbach's alpha for this study was .858.

#### Anxiety

Anxiety symptoms were measured using the anxiety subscale of the Hospital Anxiety and Depression Scale (Zigmond & Snaith, 1983) which detects anxiety symptoms in people with physical health problems. Responses range from 0 (*not at all*) to 3 (*all of the time*) on the 7‐item scale. Anxiety scores vary from 0 to 21, with high scores indicating greater levels of anxiety symptoms. Cronbach's alpha for this study was .857.

#### Cognitive function

Participants completed an interviewer administered cognitive function test at the pre‐assessment clinic (MoCA; Nasreddine *et al*., 2005). Three measures were selected to assess cognitive function. These included orientation in time, immediate recall, and verbal fluency. Higher scores indicate higher cognitive functioning.

#### Adult attachment

The Experiences in Close Relationship Scale: Relationships Structures (ECR‐RS) questionnaire (Fraley, Heffernan, Vicary, & Brumbaugh, 2011) is a 9‐item self‐report instrument designed to assess attachment patterns across different relationship domains. Patients were asked to answer the questions in relation to their romantic partner. Responses are given using a 7‐point Likert scale ranging from 1 (*strongly disagree*) to 7 (*strongly agree*). Attachment avoidance is calculated by averaging items 1–6 (e.g., ‘I don't feel comfortable opening up to this person’). Items 7–9 are averaged to compute attachment anxiety (e.g., ‘I often worry that this person doesn't care for me’). Scores range between 1 and 7; higher scores indicate higher levels of attachment anxiety and avoidance. Cronbach's alpha was .939 for attachment anxiety and .886 for attachment avoidance.

#### Social support

The ENRICHD Social Support Inventory (ESSI; Mitchell *et al*., 2003) is a 7‐item self‐report questionnaire that looks at emotional, instrumental, informational, and appraisal support. Responses range from 1 (*none of the time*) to 5 (*all of the time*), with item 7 (living with spouse) scored 4 for *yes* and 2 for *no*. Scores range from 0 to 34, with higher scores indicating higher levels of perceived social support. Cronbach's alpha for this study was .904.

### Statistical analysis

Statistical analyses were carried out using SPSS v.20 for Windows. Attachment anxiety and avoidance were treated as independent continuous variables. To test the hypothesis that attachment predicts depression and anxiety symptoms, we report data taken from baseline, then at 6–8 weeks and 1 year following CABG surgery and examine these associations using hierarchical linear regression models. Body mass index (BMI), income category, EuroSCORE, chronic disease burden, number of grafts, and social support were entered into model 1 alongside baseline depression and anxiety symptoms, when depression and anxiety symptoms at 6–8 weeks and 12 months were the dependent variables, respectively. Demographics, clinical factors, measures of socio‐economic status, and social support, as well as baseline depression and anxiety, were identified *a priori* as potential confounding variables as they have all been associated with depression and anxiety outcomes in patients with coronary heart disease (Blumenthal *et al*., 2003; Burg *et al*., 2003). Attachment anxiety and avoidance were then entered into model 2 to see whether any additional variance would be explained over and above demographic, clinical, and psychosocial factors. Adjusted *R* ^2^ and unstandardized beta values with 95% confidence intervals are reported. Preliminary analyses were conducted to ensure that no violations of the assumptions of normality, linearity, multicollinearity, and homoscedasticity.

## Results

A summary of demographic and clinical information can be seen in Table 1. The sample had an age range between 44 and 90 years, were predominantly male (87%), and overweight (BMI > 25 = 83%). The majority of patients were hypertensive, and approximately a quarter were diabetic. Most patients had on‐pump surgery. On average, participants were within the normal range for depression symptoms on the BDI; however, 32.5% of participants at baseline, 24% at 6‐ to 8‐week follow‐up, and 22% at 12‐month follow‐up scored >10. For anxiety, 80% of participants scored within the normal range at baseline, increasing to 90% at 6–8 weeks following surgery and 94% at 12 months. Overall, there were significant improvements in anxiety and depression scores for patients assessed at 6–8 weeks and 12 months compared with baseline (*p* < .05). As no interactions were found between attachment anxiety and avoidance, those results are not reported any further.

**Table 1:** Characteristics of the sample (*N *=* *155)

Characteristic	Mean ± *SD* or *n* (%)
Age (years)	68.20 ± 8.79
Female	20 (13)
BMI (kg/m^2^)	28.12 ± 3.52
Ethnicity – White British	133 (89.67)
Yearly household income	
<10,000 GBP	19 (12)
10,000–20,000 GBP	42 (27)
20,000–30,000 GBP	36 (23)
30,000–40,000 GBP	26 (17)
>40,000 GBP	32 (21)
Logistic EuroSCORE %	4.37 ± 2.97
No. of grafts	3.00 ± 1.14
Attachment anxiety	1.89 ± 1.39
Attachment avoidance	2.34 ± 1.31
Depression at baseline	8.11 ± 5.86
Depression at 6–8 weeks	6.62 ± 6.22
Depression at 12 months	6.59 ± 6.23
Anxiety at baseline	5.64 ± 4.08
Anxiety at 6–8 weeks	3.83 ± 3.64
Anxiety at 12 months	3.78 ± 3.66
Social Support Baseline	28.67 ± 5.95

Correlational analysis showed significant positive associations between attachment anxiety and avoidance with depression symptoms at all time points and anxiety symptoms at 6–8 weeks and 12 months. Social support was negatively associated with both attachment dimensions (Table 2).

**Table 2:** Correlation matrix

	1	2	3	4	5	6	7	8	9	10
Baseline										
1. Attachment anxiety		.695**	.249**	.124	−.442**	−.040	.236**	.190*	.345**	.310**
2. Attachment avoidance			.276**	.122	−.421**	−.049	.233**	.172*	.299**	.197**
3. Depression				.643**	−.286**	−.029	.456**	.445**	.499**	.433**
4. Anxiety					−.137*	−.052	.318**	.508**	.371**	.575**
5. Social support						−.153*	−.202**	−.125	−.195**	−.168*
6. EuroSCORE							.068	.046	.076	−.009
7. Depression (6–8 weeks)								.733**	.737**	.445**
8. Anxiety: (6–8 weeks)									.652**	.546**
9. Depression (12 months)										.652**
10. Anxiety (12 months)										

*p < .05; **p < .01.

### Attachment as a predictor of depression and anxiety symptoms in CABG patients

#### Depression symptoms at 6–8 weeks following surgery

We found that depressive symptoms at 6–8 weeks were significantly associated with baseline depression in model 1 (*B* = 0.473; *p* < .001; CI = 0.257 to 0.688), as was household income (*B* = −1.129; *p* = .004; CI = −1.892 to −0.366) and chronic disease burden (*B* = 2.228; *p* = .004; CI = 0.713 to 3.743). Attachment anxiety was a significant independent predictor in model 2 (*B* = 0.370; *p* = .026; CI = 0.044 to 0.696), and the variance accounted for a significant increase was from 41.4% to 43.7%. The results suggest that higher levels of attachment anxiety were significantly associated with greater depression symptom reports at 6–8 weeks following surgery. Attachment avoidance was not a significant predictor (*p* > .05).

#### Depression symptoms at 12‐month follow‐up

Baseline depression (*B* = 0.353; *p* < .001; CI = 0.142 to 0.564) and baseline anxiety (*B* = 0.351; *p* = .010; CI = 0.084 to 0.617) significantly predicted depression symptoms at 1 year (Table 3). Higher levels of attachment anxiety also predicted higher symptom reports of depression at 1 year (*B* = 0.448; *p* = .005; CI = 0.139 to 0.757). The additional variance accounted for in the final model was small (5.8%), but attachment anxiety remained a significant independent predictor of depression symptoms. Attachment avoidance was not a significant predictor (*p* > .05).

**Table 3:** Predictors of depression and anxiety 6–8 weeks and at 12 months following surgery

Model	Adjusted *R* ^2^	*F*	Variance change *p*	*B*	95% CI	*p*
6‐ to 8‐week follow‐up						
Depression						
Baseline adjusted^a^	.414	8.982	<.001			<.001
Fully adjusted model^b^	.437	8.305	.050			<.001
*Socio‐economic status (SES)*				−1.129	−1.892, −0.366	.004
*Baseline depression*				0.473	0.257, 0.688	<.001
*Chronic disease burden*				2.228	0.713, 3.743	.004
*Attachment anxiety*				0.370	0.044, 0.696	.026
Anxiety						
Baseline adjusted^a^	.482	12.685	<.001			<.001
Fully adjusted model^b^	.514	12.942	.006			<.001
Socio‐economic status (SES)				−0.722	−1.129, −0.315	.001
Social support				−0.156	0.054, 0.258	.003
Baseline anxiety				0.382	0.252, 0.512	<.001
Attachment anxiety				0.176	0.051, 0.302	.006
12‐month follow‐up						
Depression						
Baseline adjusted^a^	.266	6.833	<.001			<.001
Fully adjusted model^b^	.324	7.329	.001			<.001
Baseline depression				0.353	0.142, 0.564	.001
Baseline anxiety				0.351	0.084, 0.617	.010
Attachment anxiety				0.448	0.139, 0.757	.005
Anxiety						
Baseline adjusted^a^	.315	9.399	<.001			<.001
Fully adjusted model^b^	.389	10.289	<.001			<.001
Baseline anxiety				0.523	0.374, 0.672	<.001
Attachment anxiety				0.355	0.182, 0.528	<.001

a Baseline adjustments: BMI, smoking status, SES, EuroSCORE, grafts, chronic disease burden, baseline depression, baseline anxiety, years married, social support.

b Fully adjusted model: attachment anxiety, attachment avoidance.

#### Anxiety symptoms at 6–8 weeks following surgery

Household income (*B* = −0.722; *p* = .001; CI = −1.129 to −0.355), social support (*B* = −0.156; *p* = .003; CI = 0.054 to 0.258), and baseline anxiety (*B* = 0.382; *p* < .001; CI = 0.252 to 0.512) predicted anxiety levels at 6–8 weeks following surgery in model 1. In model 2, attachment anxiety was a significant independent predictor of anxiety symptoms (*B* = 0.176; *p* = .006; CI = 0.051 to 0.302), with higher levels of attachment anxiety predicting higher levels of anxiety symptoms (Table 3). Attachment avoidance was not a significant predictor of short‐term anxiety symptoms (*p* > .05).

#### Anxiety symptoms at 12‐month follow‐up

In model 1, only baseline anxiety levels (*B* = 0.523; *p* < .001; CI = 0.374 to 0.672) predicted higher levels of anxiety symptoms at 1 year following CABG surgery. Adding attachment into model 2 increased the variance explained by an additional 7.4%, which was a significant increase (*p* < .001). Increased levels of attachment anxiety, but not attachment avoidance, predicted higher symptom reports of anxiety (*B* = 0.355; *p* < .001; CI 0.182 to 0.528).

## Discussion

Higher levels of attachment anxiety were associated with higher levels of depression and anxiety symptoms in both short‐ and longer‐term follow‐up, independently of established risk factors, and after controlling for baseline depression and anxiety symptoms. These results correspond with existing work that has shown that attachment insecurity may lead to a vulnerability to the development and maintenance of depression and anxiety symptoms (Bifulco, Moran, Ball, & Bernazzani, 2002; Shaver *et al*., 2005). Our results also support existing work that suggests that levels of depression prevalence occur more frequently in individuals who are anxiously attached (Rholes & Simpson, 2004). The association between anxious attachment, depression, and anxiety symptoms may be a consequence of negative cognitive working models activated during times of threat (Bartholomew & Horowitz, 1991), such as a threat to health, in this case CABG surgery and recovery.

The recovery period can be quite challenging for CABG patients with chances of infection, restricted mobility, increased dependence on spouse or family members, and overall poorer QoL (Gallagher, McKinley, & Dracup, 2004; Oxlad & Wade, 2008). Depression and anxiety symptoms may be experienced more intensely by those with high attachment anxiety because of the utilization of hypervigilant strategies to identify potential threat (Kidd & Sheffield, 2005; Maunder & Hunter, 2001). Moreover, if the attachment system remains activated, distress is perpetuated, negative views of self are reinforced, and depression and anxiety symptoms may be maintained over time in those individuals (Meredith, Strong, & Feeney, 2007; Riso, Miyatake, & Thase, 2002).

It is noteworthy that avoidant attachment did not predict depression or anxiety symptoms at any time point across the study. On the one hand, this is not surprising as attachment avoidance is believed to be organized around deactivating strategies of affect regulation, which prevent the activation of the attachment system by inhibiting appraisal and monitoring of threat, the suppression of distress related thoughts, and inhibition of proximity‐seeking behaviours (Dewitte, De Houwer, Goubert, & Buysse, 2010). Largely, studies have shown that attachment avoidance is associated with low subjective reports of distress during acute stress (Kidd *et al*., 2011). Based on past findings, and the current study, it may be that attachment avoidance has a protective effect on mood during stressful events, such as CABG surgery.

The findings of this study suggest that attachment orientation may potentially be an important motivational system involved in coping with CABG surgery. Research suggests that attachment orientations are directly related to coping strategies through the appraisal of stress, and whether the individual has the resources to cope with the event (Mikulincer & Florian, 1998). In the context of CABG surgery, anxious attachment may indicate individuals with heightened stress appraisals, who feel that they are unable to cope with stress associated with the surgery unaided; however, the need for reliance on others, coupled with perceptions of low social support availability, may promote emotion‐focused coping. This is turn has been linked to greater depression symptoms (Allman, Berry, & Nasir, 2009). The coping literature has also suggested that there are instances where diverting coping strategies can be adaptive (Schmidt, Nachtigall, Wuethrich‐Martone, & Strauss, 2002), which corresponds with our findings related to those with higher levels of attachment avoidance.

Our group has previously demonstrated that depression and attachment anxiety are associated with length of hospital stay and increased levels of inflammation after CABG surgery. Depression was associated with increased levels of C‐reactive protein and longer hospital stay while attachment anxiety, but not attachment avoidance, predicted higher levels of interleukin‐6, greater sleep disturbance, and longer hospital stay following CABG surgery (Kidd *et al*., 2014; Poole, Kidd *et al*., 2014; Poole, Leigh *et al*., 2014). The current study expands on our existing work and the wider literature by providing evidence that supports attachment as a framework for understanding the aetiology and presentation of depression and anxiety symptomatology in CABG patients, which may have implications for recovery.

### Limitations

Caution needs to be taken in interpreting the data presented here as the study was limited to white, middle‐aged participants, and so we cannot generalize the results to other age or ethnic groups. There was also a preponderance of male participants in the ARCS study with 13% of the sample being female. This male majority is characteristic of the CABG surgical population more generally, with men more likely to receive a revascularization procedure than women in the United Kingdom. There may be additional bias in the results as a complete case approach for data analysis was undertaken. Moreover, we did not include any measure of threat appraisal, limiting our understanding of the initiation and sequencing of attachment activation during the study period.

We cannot say with any degree of certainty that attachment anxiety caused depression and anxiety symptoms in our CABG patients. There could be a bidirectional relationship between attachment and mood. Chronic diseases, like CAD, are likely to present significant long‐term challenges with interpersonal consequences, such as social isolation. The physical consequences of the illness could therefore influence attachment functioning. Furthermore, it is possible that those who are anxiously attached will simply over‐report symptoms to ensure that their distress is acknowledged. This seems unlikely, however, as there is a significant body of evidence indicating both a physiological and behavioural basis for attachment insecurity on health (Ciechanowski *et al*., 2003; Kidd, Hamer, & Steptoe, 2013; Maunder & Hunter, 2008; McWilliams & Bailey, 2010).

Despite these limitations, our study has a number of strengths. Firstly, attachment anxiety predicted depression and anxiety symptoms independently of established risk factors, including indices of physical health, disease severity, and baseline measures. Moreover, our sample was not very depressed with 22% of patients experiencing depression symptoms 12 months following surgery, of those around 80% reported mild depression symptoms. Equally, patients did not report much anxiety with <10% reporting mild anxiety at 12 months. Depression symptoms and major depressive disorder are understood to be part of the same psychopathology (Pignay‐Demaria *et al*., 2003). Dose‐dependent associations between depression and cardiovascular outcomes have been reported and to some extent with anxiety (Carney & Freedland, 2003; Sullivan, LaCroix, Baum, Grothaus, & Katon, 1997). This incremental association means that even mild symptoms may be detrimental to the recovery process in CABG patients. This highlights the importance of being able to identify mild symptoms as well as clinical depression, particularly as mild depression symptoms may be overlooked, or treated as cardiac‐related physical symptoms, rather than a mood disorder *per se*. Secondly, to our knowledge, we are the first to report on the ECR‐RS attachment measure in a cardiac population. This brief 9‐item scale provides a means of assessing attachment relationships without overburdening those who may be unwell in the health care environment. Thirdly, the design of our study offers the opportunity to examine naturalistic attachment processes through which depression and anxiety symptoms are experienced in a CABG population.

Finally, the results from our study correspond with the growing awareness of the importance of patient diversity and preferences in health care utilization and the impact this has on health behaviour and outcomes (Ciechanowski *et al*., 2004; Committee on Quality of Health Care in America, 2001). One advantage of using an attachment framework to understand individual variation in depression and anxiety symptoms is its potential for generalizability across medical contexts (Maunder & Hunter, 2008). It allows researchers to understand why some individuals perceive events as more threatening than others, and how these differences in threat reactivity and affect regulation may impact on mental and physical health outcomes.

Research is now needed to examine how the provision of care based around patients attachment needs could be implemented in clinical practice (Maunder & Hunter, 2009). Stepped care programmes may be one opportunity to be explored in the treatment of depression and anxiety symptoms in patients (Ciechanowski *et al*., 2004). For anxious individuals, it would be important to establish relationships with their care provider(s) and to have regular contact, whereas for those high levels of attachment avoidance, infrequent contact would be preferable. Future work should address the efficacy of such a proposal in both short‐ and longer‐term depression and anxiety outcomes in CABG populations.

In conclusion, identifying those who may be susceptible to experiencing depression and anxiety symptoms is crucial in improving recovery outcomes for CABG patients. Attachment theory provides a powerful, integrative framework for understanding how internalized working models might increase vulnerability to anxiety and depressive symptoms which may directly, or indirectly, be implicated in short‐ and long‐term recovery from CABG surgery.

## Conflict of interest

All authors declare no conflict of interest.
